# Impulse Control Disorders in Parkinson's Disease. A Brief and Comprehensive Review

**DOI:** 10.3389/fneur.2019.00351

**Published:** 2019-04-17

**Authors:** Emilia M. Gatto, Victoria Aldinio

**Affiliations:** ^1^Department of Neurology, Sanatorio de la Trinidad Mitre, Buenos Aires, Argentina; ^2^Instituto de Neurociencias Buenos Aires, Ineba, Buenos Aires, Argentina

**Keywords:** impulse control disorders, ICD, pathological gambling, binge eating, hypersexual disorder, compulsive buying, Parkinson disease

## Abstract

Impulse control and related disorders (ICDs-RD) encompasses a heterogeneous group of disorders that involve pleasurable behaviors performed repetitively, excessively, and compulsively. The key common symptom in all these disorders is the failure to resist an impulse or temptation to control an act or specific behavior, which is ultimately harmful to oneself or others and interferes in major areas of life. The major symptoms of ICDs include pathological gambling (PG), hypersexualtiy (HS), compulsive buying/shopping (CB) and binge eating (BE) functioning. ICDs and ICDs-RD have been included in the behavioral spectrum of non-motor symptoms in Parkinson's disease (PD) leading, in some cases, to serious financial, legal and psychosocial devastating consequences. Herein we present the prevalence of ICDs, the risk factors, its pathophysiological mechanisms, the link with agonist dopaminergic therapies and therapeutic managements.

## Definition

Impulse control and related disorders (ICDs-RD) encompass a heterogeneous group of disorders that involve pleasurable behaviors performed repetitively, excessively, and compulsively (1–8).

The common key symptom in all of these disorders is the failure to resist an impulse or temptation to control an act or specific behavior (1, 3, 9), which is ultimately harmful to oneself or others and interferes in major areas of life functioning (1, 3, 6, 10, 11).

The American Psychiatric Association's Diagnostic and Statistical Manual (DSM-5) included impulse control disorders (ICDs) in the chapter of “Disruptive, Impulse-Control, and Conduct Disorders” as a dysregulation of self-emotional and behavioral control (8).

ICDs have recently been sub-classified as ICD groups and ICD-related disorder (ICDs-RD) groups (1, 3, 6, 7).

The major symptoms of ICDs include pathological gambling (PG), hypersexuality (HS), compulsive buying/shopping (CB) and binge eating (BE) (1–4, 8, 9, 12–21).

However, PG was moved from the category of ICDs to a new category of “Substance-Related and Addictive Disorders” in the DSM-5 (1–3, 6, 7), taking into account the similarities to drug addiction (risk factors, clinical features, cognitive changes, neurobiological substrates, and treatment approaches) (2, 6). This modification highlights the variability of reward-driven behaviors (2, 6, 16, 22).

The spectrum of ICDs-RD also includes punding, hobbyism, walkabout, hoarding, and compulsive medication use.

ICDs and ICDs-RD have been included in the behavioral spectrum of non-motor symptoms in Parkinson's disease (PD), leading in some cases to serious financial, legal and psychosocially devastating consequences with a greater impact on the quality of life. Moreover, in recent years we have noticed that PD patients are at increased risk of developing more than one of the major ICDs.

Along these lines, although it is not the focus of the present paper, some authors have suggested that the increased drive or motivation to certain behaviors cannot be harmful but rather beneficial (1). Therefore, it remains under discussion whether artistic productivity or hypercreativity should be included in ICDs or in ICDs-RD, or if it might represent an innate–skill that emerges in PD patients on dopaminergic therapy (8, 12, 13, 23, 24).

## Component Aspects

Three main aspects that characterize ICDs groups and ICD-related disorders in relation to reward-driven activities are:

1. The presence of impulsive aspects (lack of forethought or consideration of consequences) (1, 3, 9).
2. The presence of compulsive aspects (repetitive behaviors with a lack of self-control) (1, 3, 9).
3. A negative or harmful behavior to oneself or to others (1, 3, 6).

The four major ICDs include:

Pathological Gambling (PG) characterized by an excessive and uncontrollable “preoccupation with gambling and the excitement that gambling with increasing risk provides” despite financial loss and social problems (3, 7, 22, 25–27). PG was one of the earliest recognized ICDs in PD (3). It was recently moved to the category of “Substance-related and addictive disorders” in the DMS-5, since substance abuse and PG activate brain reward areas and this bears similarities to drug addiction (7, 28).

Hypersexual disorder (HS) included in “The Sexual and Gender Identity Disorders Workgroup” of DSM-5 (7). It could be described as an excessive amount of time consumed by sexual fantasies and by planning for engaging in sexual behavior which interferes with important activities and obligations in ordinary life (3, 7). Other behaviors that might often occur are fetishism and voyeurism (7). As in substance abuse, patients with HS pursue a short-term reward and may develop tolerance and withdrawal-like syndromes (7). This condition is more common among adult men, and it may additionally occur with erectile failure (6, 7, 9, 17, 29).

Binge eating has been included in “Feeding and Eating Disorders” in DSM-5 (3, 6, 7). It is “a persistent disturbance of eating or eating-related behavior that results in the altered consumption of food, which significantly impairs physical health or psychosocial functioning” (7). The specific criteria proposed are:

1. Episodes of recurrent binge eating in the absence of any maladaptive compensatory behaviors.
2. Sense of lack of control over eating during the episodes.
3. Intake, in a discrete period of time (within any 2 h period), of an amount of food that is much larger than most people would eat in a similar period of time under normal circumstances.

The difference between binge eating and bulimia is that the former tends to be fluctuating while the latter is permanent (3, 7).

Compulsive buying (CB) is characterized by a constant urge to buy that leads to senseless contraction of debts with continuous delay of payment until a catastrophe clears the situation. As other ICDs, the repetitive loss of control over spending and the negative emotional state that emerges when not buying resemble substance use disorders (3, 7).

A prevalence of 5.8% in the general population at risk of CB is described (1, 3).

## ICD-Related Behaviors (ICDs-RD)

ICDs-RD are classified as related behaviors that have a contrast clinical presentation with respect to the four major ICDs. However, the biological link between both conditions may be identified in the dysregulation or inappropriate regulation of the reward pathways in the mesocorticolimbic network (22, 30). ICDs-RD is characterized by repetitive perseverative behaviors that appear to be more closely linked to pulsatile drugs, such as levodopa or intermittent apomorphine therapy rather than dopaminergic agonist (DA) *per se*.

ICDs-RD include the following:

1. Dopamine dysregulation syndrome (DDS) is a drug addiction-like state characterized by a compulsive and excessive desire for use of high potency and short-acting dopaminergic medication (L-dopa, subcutaneous apomorphine) (1–4, 6–8, 12, 13, 15, 17–22, 30, 31). DDS is more frequent in early-onset male PD patients with history of mood disorders and family history of psychiatric disorders (26, 31).
2. Punding is characterized by repetitive, purposeless behaviors and excessive preoccupation with specific items or activities, collecting, arranging or taking objects apart (1–4, 6–8, 12, 15, 17–21, 26, 32). It has been reported to occur frequently in conjunction with DDS (32).
3. Hobbyism pertains to higher-level repetitive behaviors (sports, artistic endeavors) (1, 2, 4, 6, 8, 15, 17–21).
4. Walkabout is excessive aimless wandering (1, 2, 4, 7, 8, 12, 15, 17, 19–21, 26).
5. Hoarding is the acquisition of and failure to discard a large number of items with no objective value (1–4, 6, 7, 12), (8, 15, 18, 21).

## Epidemiology

### ICD in the General Population

The prevalence of ICDs in the general population, which has been underestimated, shows a wide range with variability according to different populations: from 0.2 to 5.3% (1). This enormous variability may be explained not only by different genetic, ethnic and cultural backgrounds, but also by the instruments used to assess these symptoms in the population (3, 18–20) (Table 1).

**Table 1:** Shows the estimated prevalence of each of the four major ICDs.

**ICDs**	**General population**	**Dominion study** **ICD subgroup**	**ICARUS study (at baseline, use QUIP)** **ICD subgroup**	**The drug interaction with genes in Parkinson's disease DIGPD study (ICD at baseline)**
Total	0.2–5.3%	17.10%	28.6%	19.7%
Age (mean SD)	N/A^*^	60.2 (8.1)	63.6 ± 9.5	58.5 (8.9)
UPDRS III score (mean SD)		N/A	14.1 ± 5.89	18.8 (9.4)
Cognitive scores MMSE adjusted total score			27.9 ± 1.62	28.4 (1.7)
Disease Duration		7.1 (3.8–10.8)	6.9 ± 5.19	3.1 (1.4)
Median dopamine agonist LEDD		300 mg	N/A	211.1 (118.0)
Median levodopa LEDD associated DA		450 mg		N/A
Median levodopa LEDD without a dopamine agonist		621 mg		263.4 (230.7)
Compulsive Buying	5.8% (2–8)	5.7%	6.5%	4.6%
Pathological Gambling	0.4–1.1%	5%	5.30%	3.9%
Binge eating	2%	4.3%	9.9%	10.5%
Hypersexual disorder	3–6%	3.5%	9.7%	8.5%
References	(1)	(29)	(19)	(33)

N/A

* *: non available or Non applicable*.

Although the ICDs were initially reported in PD patients on DA therapies, some studies report the occurrence of ICDs in the general population and in novo PD patients (10, 11, 34). It is still under discussion whether PD biology could be a risk factor for ICDs (35).

### ICD in *de novo* PD

As mentioned above, it remains under discussion whether or not PD itself confers an increased risk for developing ICDs (35). Identifying the frequency of this disorder in novo PD patients could contribute to resolving these questions (1). A recent study analyzing data from the Parkinson's Progression Markers Initiative failed to demonstrate an increased risk for the development of ICDs or ICDs-RB in PD patients in the absence of treatment. Nevertheless, some symptoms suggestive of ICD have been reported in 20% of newly diagnosed, untreated PD patients with respect to the appropriately matched controls (36). In recent years, imaging studies have offered relevant insight to this debate (35). However, at the moment, results remain controversial over whether PD itself constitutes a risk factor for the development of ICDS or ICDs-RD (1, 3, 6).

### ICDs-RD in PD in Different Populations

ICDRs continue to be under-recognized and under-managed in clinical practice. Determining the true frequency of ICDs in the health population, in PD *de novo* patients, and in PD patients with and without DA agonist therapies in different populations represents a significant challenge since a number of variables must be analyzed, including assessment tools, DA dose, DA formulations, years of disease, as well as cultural and other factors. Moreover, in many cases more than one ICD has been identified (29). In Table 2 we present a summary of various studies conducted to assess the presence of ICD behaviors over different periods of time and evaluate the risk factors and clinical characteristics.

**Table 2:** Shows different epidemiologic studies.

**References**	**Study**		**Type of study**	***N*** **participants**		**Scales**			**Age (mean/SD) years**		**Disease duration (mean/SD) years**		**Motor scores (mean/SD)**		**Levodopa or Levodopa equivalent LEED Dopamine agonist L-dopa dose equivalent DA-LEDD**		**Results**
	**Name of the study**	**Geographic Distribution**		**PD**	**Controls**	**Motor**	**ICDs**	**Others**	**PD ICDs**	**PD non** **ICDs**	**PD ICDs**	**PD non ICDs**	**PD ICDs**	**PD non ICDs**			
Driver- Dunckley et al. (37)	Initial	United States	Retrospective database	1,884	N/A	H&Y, UPDRS	N/A		57.2 (30-72)	N/A	11.6 (4–22)	N/A	mean H&Y stage 2.5	N/A	Mean dose LEDD 883.4 mg/day	Pramipexole 4.3 mg/day Pergolide 4.5 mg/day	PG can occur as the PD progresses, appears with an increase in DA therapy and resolves reduction
Maia et al. (38)	N/A	Brazil	Case/control study	100	100	UPDRS mHYS	Y-BOCS,	SEADLS	62.2 ± 11.9 Total PD		N/A	N/A	Total UPDRS mean 40.28 ± 20.6		N/A		OCD are NOT MORE frequent in PD patients
Weintraub et al. (29)	DOMINION Study	United States and Canada	Cross-sectional, multicenter	3,090	N/A	H&Y	Massachusetts Gambling Screen, MIDI		60.2 (8.1)	64.4 (7.8)	7.1 (3.8–10.8)	6.5 (3.7–10.6)	2.0 (2.0–2.5) H&Y	2.0 (2.0–2.5) H6Y	Pramipéxole 3.1 mg (SD, 1.7 mg) and LEDDs 306.9 mg (SD, 168.2 mg) Ropirinole 11.1 mg (SD, 6.6 mg) and LEDDs 277.9 mg (SD, 164.9 mg) Pergolide 2.9 mg (SD, 1.7 mg) and LEDDs 286.6 mg (SD, 169.3 mg)		DA treatment in PD is associated with 2- to 3.5-fold increased odds of having an ICD
Joutsa et al. (59)		Finland	Cross-sectional.	575	N/A		South Oaks Gambling Screen, QUIP,	BDI.	64 (range 43–90) total PD		6 (< 1–29) years Total PD		N/A	N/A	Total L-Dopa was 561 (26–3,230) mg DA LEDD was 160 (105–210) mg		There is a high proportion of patients with PD with ICDS. Prevalence of PG in PD is 7 times higher than general population. Depression associated with all ICDS.
Sarathchandran et al. (39)		India	Case/control study	305	234	H&Y UPDRS	MIDI, DSM IV, BIS, BDI	Eysenck personality inventory; Anxiety and Depression Scale, PDQ-39	54.6 ± 9.9	59.6 ± 9.8	8.2 ± 4.9	7.3 ±4.8	H&Y ON 2.0 ± 0.5 UPDRS-III ON 18.7 ± 9.2	H&Y in ON 1.9 ± 0.5 UPDRS-III ON 18.5 ± 8.8	PD without ICD LEDD: 448 ± 280 mg; L-Dopa:326.2 ± 31.9 mg PD with ICD LEDD: 590 ± 364.8 mg; L-Dopa: 373.4 ± 68.5 mg		Revealed a relatively higher frequency of ICD-RBs
Rodríguez-Violante et al. (40)		Mexico.	Case/control study	300	150	MDS-UPDRS H&Y	QUIP-RS		58 ± 14.1	63 ± 12.5	N/A	N/A	MDS- UPDRS part III 31 ± 15.9; H&Y: 2.2 ± 0.6	MDS-UPDRS part III 32.8 ± 17; H&Y 2.3 ± 0.8	PD with ICD group LEDD 638 ± 448.5 mg; DA-LEDD: 147.4 ± 123.3 mg PD without ICD LEDD: 561.3 ± 417.4 mg; DA-LEDD: 97.1 ± 124.9 mg		ICD significantly more frequent in PD than controls subjects. lower overall frequency and distinct pattern of ICDs related with socioeconomic differences
Ramírez Gómez et al. (5)		Argentina, Colombia, Ecuador	Multicenter. Structured Clinical Interview. cross-sectional	255	N/A	UPDRS; H&Y	QUIP, QUIP-RS; CISI-PD		58.6 (SD, 11.11)	N/A	4	10	Mean UPDRS 10	Mean UPDRS 33	N/A		ICD in Latin American PD > Anglosaxon population
Rizos et al. (41)		UK, Spain, Denmark and Romania	Multicenter Retrospective and prospective survey based on medical records and clinical interviews.	425	N/A	H&Y	NMS Questionnaire		62.7 (42–85)		7.0 (0–24)	N/A	H&Y: 3.0 (1.0–5.0)	N/A	N/A		Relatively low rate of ICDs with long-acting or transdermal DAs.
Vela et al. (17)		Spain	Multicenter study, Cross-sectional, case/control study	87	87	UPDRS; H&Y	QUIP	BID, EuroQol, PDQ-39	48 (44–52)	46 (42–52)	7 (3–11)	3 (1–10)	Mean UPDRS III: 16 (10–22); H&Y:2 (2–2)	Mean UPDRS III 17 (11–24); H&Y 2 (1–2)	LEDD 300 (0–600) mg DA LEDD 210 (99–300) mg		ICBs are much more prevalent in early onset PD patients vs. health controls Associated with DA intake, depression and a worse QoL
Erga et al. (20)	Norwegian ParkWest Study	Norway	Multicenter Cross-sectional study, Semistructured Clinical interviews, cases and controls	125	159	UPDRS; H&Y	QUIP	MMSE, Stroop test, Semantic verbal fluency test, CLVT-II, VOSP, NPI, MADRS, Epworth Sleepiness Scale (PDSS-2	67.9 (7.7)	71.4 (9.8)	7.4 (1.6)	7.4 (1.9)	H&Y: 2.2 (0.5); Mean UPDRS III: 23.8 (10.5)	UPDRS motor score 22.7 (10.6). H&Y: 2.2 (0.6)	PD without ICD LEDD: 408.7 ± 266.7 mg; DA LEDD:289.5 ± 150.0 PD with ICD LEDD: 505.2 ± 279.1; DA LEDD: 293.7 ± 132.4		Patients with PD treated with DA, have increased odds of having ICBs compared with age- and gender-matched controls.
Biundo et al. (42)	ALTHEA study	Italy	Multicenter	251	N/A	H&Y, UPDRS; UDysRS	QUIP-RS; BDI	MoCA;BDI-II	ICD-RBs below cut-off 66.5 6 10.2 ICD-RBs above cut-off 63.5 6 9.9	67.2 ± 9.4	ICD-RBs below cut-off 52.7 ± 61.1 (months); ICD-RBs above cut-off 148.0 ± 64.5 (months)	140.2 ± 68.21 (months)	ICD-RBs below cut-off UPDRS III: 11.9 ± 7.1 ICD-RBs above cut-off UPDRS III: 12.2 ± 7.4	UPDRS III: 11.8 (6.9)	No ICD-RBs LEDD 971.0 6 ± 401.1 mg; DA-LEDD 147.0 6 ± 162.7 mg ICD-RBs above cut-off LEDD 1,016.4 6 ± 418.3 mg; DA-LEDD 133.1 ± 129.0 mg		>50% of PD patients with dyskinesia have ICDs and RBDs. Severity is associated with Dopaminergic therapy total dose
Zhang et al. (4)		China	Xin Hua Hospital	142		H&Y, UPDRS, the scale for freezing of gait	QUIP.	MMSE, NMS, RBDQ-HK, HAMA, HAMD, PDQ- 39	65.55 ± 7.43	69.67 ± 8.16	7.76 ± 5.90	5.22 ± 5.23	Mean UPDRS: 20.18 ± 11.56; H&Y: 2.32 ± 0.99	Mean UPDRS: 18.93 ± 12.82; H&Y: 2.21 ± 0.77	Total LEDD, mg PD without ICD:329.82 ± 340.65 mg PD with ICD: 522.06 ± 412.46 mg		ICD and RBD commonly found in Chinese PD patients. Independent factors associated with ICRDs: Earlier onset, dose of DA, severe cognitive impairment; dyskinesia.
Antonini et al. (19)	ICARUS Study	Italy	Prospective, non-interventional, multicenter	1,069 DA alone L-Dopa alone L-Dopa + DA		H&Y, UPDRS	mMIDI; QUIP	NMSS, PDSS-2, PD-CRS, PDQ-8, BDI-II, FAB and three items of NPI-3: delusions, hallucinations and apathy/ indifference.	63.6 ± 9.5	66.6 ± 9.3	6.9 ± 5.19	5.8 ± 4.92	H&Y: 2.0 ± 0.70; Mean UPDRS III: 14.1 ± 5.89	H&Y:2.0 ± 0.63; Mean UPDRS III: 14.2 ± 7.09	N/A		Prevalence of ICD was relatively stable throughout the 2-years follow-up. No differences between patients receiving DAs and those on L-Dopa. No differences between PD with or without ICD in motor symptoms severity and cognitive function.
Corvol et al. (33)	DIGPD	France	Multicenter, face to face semistructured interviews.	411	N/A	MDS-UPDRS (parts I–IV) H&Y	MDS-UPDRS part I	Mini-Mental State	58.5 (8.9) at Baseline	63.3 (9.8) at Baseline	3.1 (1.4) at Baseline	2.5 (1.5) at Baseline	Mean UPDRS III: 18.8 (9.4)	Mean UPDRS III: 20.5 (10.5)	Baseline NO ICD LEDD 235.7 ± 181.1; DA-LEDD: 145.0 ± 99.1 ICD LEDD:263.4 ± 230.7; DA-LEDD: 211.1 ± 118.0		5-years cumulative incidence of ICDs ≈46%. ICDs: strongly associated with DA use and dose-effect.

*Anxiety and Depression Scale; BDI-II, Beck Depressive Inventory; CISI-PD, Clinical Impression of Severity Index for Parkinson's Disease; CLVT-II, California Verbal Learning Test II; Epworth Sleepiness Scale; Eysenck personality inventory. FAB, Frontal Assessment Battery; H&Y, Hoehn & Yahr stage; HAMA, Hamilton Anxiety Scale; HAMD, Hamilton Depression Scale; MADRS, Montgomery and Asberg Depression Rating Scale; Massachusetts Gambling Screen; MDS-UPDRS; mH&Y, modified Hoehn & Yahr stage; mMIDI, modified versión of the Minnesota Impulsive Disorders Interview; MMSE, Mini Mental State Examination; MoCA, Montreal Cognitive Assessment; NMSS, Non-Motor Symptom Scale; NPI-3, Neuropsychiatric Inventory; PD-CRS, Parkinson's Disease-Cognition Rating Scale; PDQ-39, 39-item Parkinson's Disease Questionnaire; PDQ-8, Parkinson's Disease Questionnaire-8 items; PDSS-2, Parkinson's Disease Sleep Scale-2; QUIP, Questionnaire for Impulsive-Compulsive Disorders in Parkinson's Disease; QUIP-RS, Questionnaire for Impulsive-Compulsive Disorders in Parkinson's Disease Rating Scale; RBDQ-HK, REM Sleep Behavior Disorder Questionnaire Hong Kong; SEADLS, Schwab and England Activities of Daily Living Scale; South Oaks Gambling Screen; UDysRS, Scale Unified Dyskinesia Rating Scale; UPDRS, Unified Parkinson's Disease Rating Scale; VOSP, Visual Object and Space Perception Battery; Y-BOCS, Yale Brown Obsessive Compulsive Scale; LEDD, L-dopa-equivalent daily dose; DAED, dopamine agonist-equivalent daily dose; DA, Dopamine Agonist; ICD, impulse control disorders; RBDs, Related Behavior Disorders*.

### Assessment Tools

Several instruments have been developed to assess and identify ICD symptoms in PD, some of which are summarized in Table 3.

**Table 3:** Assessment tools.

**Tools**	**Objectives**	**Brief description**	**Translated into other languages other than English**		**Self-administered**		**References**
			**Yes**	**No**	**Yes**	**No**	
Questionnaire for impulsive-compulsive disorders in PD (QUIP)	To screen ICRDs in PD patients.	Most commonly used, validated, self-report screening tool to assess ICDs	+ German, Italian		+		(43)
QUIP rating scale (QUIP-RS)	To screen ICDs in PD patients	Rates severity of the ICDs and provides a measure of change over time	+ German, Italian, Spanish		+		(44), (45, 46)
Minnesota Impulsive Disorders Interview (MIDI)	To assess the degree of impulsivity related to compulsive behavior	A questionnaire to assess the presence of impulsive–compulsive behaviors associated to dopamine replacement therapy in PD.		+		+	(47–49)
Dopamine Dysregulation Syndrome-Patient and Caregiver Inventory (DDS-PC)	To screen ICRDs in PD patients	Questionnaire to assess the presence of several ICD behaviors associated to DDS in PD, for both self-report and caregiver's report, to uncover eventual discrepancies.		+	+		(50)
Movement disorders Society UPDRS, included a single item for DDS	Not valid as an assessment tool for ICDs	The MDS-UPDRS contains questions/evaluations, divided in three domains scoring 18 items of motor, behavior and daily activities		+	+		(51)
Barrat Impulsiveness Scale (BIS)	To assess impulsivity in PD patients.	High reliability and high predictive validity to assess high risk behaviors including symptoms of conduct disorders, attention deficit disorders, substance abuse and suicide attempt.	+ Brazilian Portuguese, Spanish, dialectal Arabic		+		(52–55)
Ardouin scale of behavior in Parkinson's disease	To assess neuropsychiatric features in PD patients	Specifically designed for asses mood and behavior, quantifying changes related to Parkinson's disease, to dopaminergic medication, and to non-motor fluctuations		+		+	(56)
Structured Clinical Interview for Obsessive-Compulsive Spectrum Disorders (SCID-OCSD)	To determine the presence of a range of ICDs.	A structured clinician-administered interview for the diagnosis of putative OCSDs		+		+	(57)
Parkinson's Impulse Control Scale (PICS)	To rate severity of ICD in PD patients.	A brief, clinician-rated screening tool that assess the intensity and impact of a wide range of ICBs common in PD		+		+	(58)

*QUIP, Questionnaire for impulsive-compulsive disorders in PD; QUIP-RS, QUIP rating scale; MIDI, Minnesota Impulsive Disorders Interview; DDS-PC, Dopamine Dysregulation Syndrome-Patient and Caregiver Inventory; BIS, Barrat Impulsiveness Scale; SCID-OCSD, Structured Clinical Interview for Obsessive-Compulsive Spectrum Disorders; PICS, Parkinson's Impulse Control Scale*.

### Risk Factors

Several studies have been conducted to identify the risk factors for ICD development in PD patients (8). They include:

+ Demographic: young patient, male gender, unmarried (3–8, 14–21, 24, 27, 29, 59, 60).
+ Treatment related: although ICDs have been reported to be associated to different drugs, such as L-dopa, amantadine and rasagiline, DA intake appears as the major risk factor for ICDs (1–5, 7, 8, 13–15, 17–22, 27, 29, 59, 60).
  Prevalence of ICDs was compared among different DA drugs (pramipexole, ropirinole) and between extended releases or immediate formulations (1, 3, 6, 29, 60). However, controversial findings from preliminary reports suggest that long-acting DA and patch or pump formulations may reduce the risk for ICDs (8, 15, 61).
  It remains under discussion whether there is an association between ICDs and DA dose. The same controversial results were reported regarding DA treatment duration, higher daily dose and DA higher peak dose (3, 7, 29, 60).
+ Personal or family history: history of cigarette smoking, drug abuse, depression, apathy, REM behavior disorders (RBD), tea, coffee and mate consumption, positive personal or family history of alcoholism or gambling, and impulsive or novelty-seeking traits increase the risk for ICDs and their predictors (2–8, 14, 16–18, 29, 59, 60).
+ PD onset and related ICDs: prevalence increases over time, while ICDs tend to occur in the first years of the disease. Early PD onset and the presence of motor complications of PD may predict a higher risk for ICDs (4–8, 13, 14, 16–18, 21, 24, 29, 60).
+ Cultural factors: it remains to be determined if cultural factors may increase the risk for ICDs and ICRDs. Some authors suggest that cultural factors probably contribute not only to the prevalence of ICD but also the type of ICD (7, 17). One classic example in this field was provided by the DOMINIO study that suggests that living in the United States of America may be an independent risk factor for ICD development (1, 6, 29).
+ Deep Brain Stimulation (DBS): the relationship between ICDs and DBS remain under discussion. Initial studies reported improvement in ICDs after DBS, while subsequent studies showed ICD exacerbation (1, 6, 22, 60, 62).
  DBS of the subthalamic nucleus (STN) is an effective, widely used treatment for motor fluctuations or disabling dyskinesias in PD (63).
  STN-DBS has been identified as an independent risk factor for ICRDs; however, the reduction of dopamine agonist dosage after STN-DBS could improve or decrease ICD occurrence (6, 7, 22, 60, 62).
  On the other hand, several studies suggest that DBS may contribute to impulsivity, excessive reward seeking and ICDs. Consistent with this hypothesis, PD patients without ICDs showed impulsive decision making when DBS is turned on (7, 60, 62, 64).
  To explain these controversial findings, it has been hypothesized that STN stimulation plays a role in dynamic aspects of impulse and inhibitory control (22, 60).
+ Personality, Neuropsychiatric symptoms and Cognition in ICDs: a higher level of neuroticism, ineffective coping skills, and lower levels of agreeableness and conscientiousness in PD patients with ICDs has been reported (3). Early onset PD patients constitute a high risk population for ICDs with a self-assertive/antisocial and reserved personality and somatization traits (22).
  A large constellation of comorbid affective symptoms and behavioral traits have been reported in PD with/or at risk for ICDs including depression, anxiety, novelty seeking, impulsivity symptoms and anhedonia (2, 62, 65, 66). Interestingly, in PD patients with ICDs, apathy could be noticed during withdrawal from dopamine replacement therapy (DRT). Impulsivity and apathy are two major comorbid syndromes of PD that may represent two extremes of a dysexecutive and behavioral spectrum involving dopamine-dependent cortico-striato-thalamo-cortical networks (64).
+ Cognition: controversial data have been identified in cognitive battery tests between PD patients with and without ICD (8, 36); the first group presents values lowered in some tests that evaluate the frontal lobe, but did not find significant differences in executive functioning (14, 67). Cognitive flexibility and ability to plan is altered in patients with ICD (8).
  Visuo-spatial working memory and reward-punishment learning impairments have been reported in different studies; however, many results could not be replicated (6, 17).
  Interestingly, patients with ICDs showed a more immediate reward response and greater choice impulsivity leading to increased risk behavior (6).
  When the cognitive performance was compared according to the type of ICD it was found that patients with HS showed greater general cognitive impairment, including lower performances on learning tests and were more impaired on the Stroop test and memory tasks than were patients with PG (8, 68). However, another study found no differences in the executive functions of patients with PD and PG (69).
+ Genetics: genetic factors have been involved in ICDs in PD. Although heritability was estimated to be 57%, consensus remains a challenge and data need to be replicated in large cohorts from different populations (16). A large number of single nucleotide polymorphisms (SNP) in dopaminergic, glutamatergic, serotonergic, and opioid neurotransmitter systems has been reported as a candidate that improved predictability of ICDs when compared with clinical risk factors (2, 6, 9, 16, 21, 70). Recently, an association of OPRM1 rs1799971 was identified, a gene encoding the mu opioid receptor with ICDs. This gene is central to pain control as well as drug reward and addictive behaviors (70).

In Table 4 we present the genetic factors reported to be related to ICDs.

**Table 4:** We present the genetic factors reported to be related to ICDs.

**Receptor types**	**Genotype**	**Associations**	**References**
Dopamine	DRD1rs4867798, rs4532, rs265981	Increased risk of ICDs	(9, 71, 72)
		PD: punding and hobbyism behaviors, ICDs	
		Non-PD: ICDs, neuropsychiatric disease, problem gambling, addiction, and cognitive functioning in non-PD population	
	DRD2 Taq1A Dopamine transporter (DAT1)	No association	(9)
	DRD2/ANKK1 rs1800497	Increased risk of ICDs	(9, 16, 65, 71)
	Dopa decarboxylase (DDC) rs 3837091; rs 1451375	Stronger predictor f ICDs	(16)
	D3Rp.S9G	ICDs and levodopa-induced dyskinesias	(2, 5, 6, 9, 18, 21, 65, 73)
		Stronger predictor of ICDs	
Glutamate	Grin2B rs7301328	Increased risk of ICDs	(2, 5, 6, 9, 16, 71)
Monoamine Transporters	COMT gene Val158 Met	No association	(9, 65)
	COMT rs4646318	No association	(9)
Opioid	OPRK1 rs702764	Stronger predictor f ICDs	(9, 16, 65, 72)
Serotonine	Hydroxytryptamine receptor HTR2A rs6313	Stronger predictor f ICDs	(2, 6, 8, 9, 16, 18)

Interestingly, the ICARUS study, the largest prospective observational study in an Italian population, contributes to the identification of additional risk factors that include non-motor symptoms (mood and sexual function), mood symptoms (depression), sleep disorders and a low level of quality of life (19).

### +Other Risk Factors

Recently, the overexpression of ΔFosB, a transcriptional regulator involved in addiction induced by drugs of abuse and in many types of compulsive behaviors has been reported to be associated with L-dopa induced dyskinesia and to be triggered by pramipexole (60).

The ΔFosB overexpression was identified in the nucleus accumbens (NA) and the striatum (brain regions important for addiction) of healthy and DA-lesioned rats exposed to pramipexole and found to be NMDA receptor dependent. These findings suggest that enhanced ΔFosB expression may represent the strongest predictor of PD patients at risk of ICDs (27, 60).

## Pathophysiology

Although an extensive number of studies have focused on the pathophysiologic mechanisms of ICDs in PD, these remain to be clarified (2, 9). Classically, the appearance of impulsivity in PD has been attributed to neuronal dopaminergic degeneration, facilitating ICD occurrence in dopamine replacement therapies (8).

Nevertheless, in recent years, evidence has suggested a complex multifactorial mechanism beyond the dopaminergic corticostriatal networks, including a complex serotoninergic and noradrenergic interaction. Further investigation is required (9).

## Dopaminergic Theory

Dopaminergic receptors, Dopamine 1 receptor 1 (D1R) (D1 and D5) and Dopamine 2 receptor (D2R) (D2, D3, D4) types possess contrasting roles with inhibitory and excitatory signaling, respectively. These contrasting roles are present not only in the nigro-striatal pathway but also in the mesolimbic and mesocortical circuits. The pathways link cortical and subcortical regions [prefrontal cortex (PFC), ventral striatum, VTA and amygdala]; both circuits are implicated in reward learning and executive decision making or reinforcement behaviors, respectively (6, 22, 74).

Anatomical regions involved in ICDs:

1. Planning and judgment areas: caudal orbitofrontal cortex, ventromedial prefrontal cortex (PFC).
2. Reward system: ventral striatum (VS-nucleus accumbens [NA]).
3. Conditioned responses and emotional processing: amygdala.
4. Medial dorsal and anterior nucleus of the thalamus (6, 75).

In PD with ICDs a marked decrease ventrostriatal D3R-binding has been reported, while experimental PD models have shown an increase in DA levels in the NA associated to bilateral nigrostriatal DA denervation (64, 76). These findings, of a diminished striatal D2/D3 receptor level and an increase in mesolimbic DA tone, lead to an imbalance in the cortico-accumbens network implicated in reward signaling and behavioral changes (64, 77, 78). Moreover, the dopaminergic mesocorticolimbic system provides a role for shift behavior in response to changing stimulus-reward contingencies (64).

In this scenario, the tonic “overdosed” by D2/D3 receptor agonists in the mesocorticolimbic circuit could contribute to suppress, through the impairment of top-down inhibitory control from prefrontal cortical area (PFC) inputs to the ventral striatum, reward-related learning and induce compulsive, perseverative behavior through the direct D1 receptor pathway (6, 9, 22).

Dopaminergic agonists (DA) show a high D3R affinity in the mesolimbic system (6, 7, 9, 60). In effect, DA therapy, acting on the depleted dorsal striatum (involved in the sensory-motor circuit) and a relatively intact ventral striatum, induces a reduction of inhibitory response and impulse control by the reduction of activity in the lateral orbitofrontal cortex, the rostral cingulated zone, the amygdala, and in the external pallidum (6, 7). Therefore, PD patients on DA are not only at high risk for ICDs but also demonstrate greater choice impulsivity, shorter reaction time and increased risk taking (6, 79).

The D1 receptor family localize in the direct pathway of reward-based behaviors. Stimulation increases the activity of striatal projections to the nucleus accumbens/ventral striatum, while D2 receptors elicit suppression of the cortico-accumbens network (6, 22, 80).

## Neuroimaging in PD Patients With ICDs

In recent years neuroimaging, particularly that which is focused on the dopaminergic system, has significantly contributed to the knowledge of neurobiological factors for ICDs (2, 7, 8, 81, 82) (see Tables 5A–D).

**Table 5A:** Structural MRI.

**Study objectives**	**Participants**			**Results**	**References**
	**PD ICD/RBDs**	**PD No ICD/RBDs**	**Controls**		
To demonstrate morphometric changes	X	X	X	No significant changes PD + ICD vs. PD-ICD	(83)
To measure brain cortical thickness and subcortical volumes, and to assess their relationship with presence and severity of symptoms, in PD patients with and without ICDs.	x	x	x	In ICD+: Significant cortical thinning in right superior orbitofrontal, left rostral middle frontal, bilateral caudal middle frontal region, and corpus callosum and reduced volume in right accumbens and increase in left amygdala in ICD	(84)
To identify Neuroanatomical abnormalities in PD patients with PG	Pathological Gambling (PG)	X	X	Gray matter loss in bilateral Orbitofrontal-cortex in PD-PG vs. PD-CNTR correlated with increase of gambling symptoms in PD-PG	(85)
To assess brain structural and functional alterations in patients PD-ICB vs. controls and PD no-ICB	x	x	x	Cortical thinning in left pre-central and superior frontal cortices, as well as decreased FA of the left uncinate fasciculus and parahippocampal tract; increased mean, radial and axial diffusivity of the left parahippocampal tract and right pedunculopontine tract; increased mean and radial diffusivity of the genu of the cingulate cortex and right uncinate fasciculus.	(86)
To assess whether a functional dysregulation of the habenula and amygdala (modulators of the reward brain circuit), contributes to PD punding.	X Punding	x	x	Cortical thinning of right inferior frontal gyrus compared to controls and PD-without punding	(87)
To investigate structural abnormalities in mesocortical, limbic cortices and subcortical structures in PD ICDs.	x	x	x	Volume loss in the nucleus accumbens of PD patients. PD-ICD showed significant increased cortical thickness in rostral anterior cingulte cortex and frontal pole compared to PD-without ICD. Increased cortical thickness in medial prefrontal regions in PD-ICD	(88)
To determine morphometric changes as predictors of ICB in de novo PD	x	x	x	No significant morphometric changes in PD-ICD and PD-without ICD before and after onset of ICD.	(89)
To better understand the neural basis of ICDs in PD	x	x	x	PD-ICD patients showed a reduced gray mater volume in External Globus Pallidus compared to PD-without ICD	(90)
To investigate gray matter (GM) and cortical thickness (CTh) changes in PD with and without ICDs.	x	x	x	Increased cortical thickness in anterior cingulate cortex, orbitofrontal cortex in PD-ICD.	(91)
Morphometric Changes in PD punding patients	Punding	X	X	Significant cortical thinning in dorsolateral prefrontal cortex in PD-punding. Cortical thinning in PD-punders localized in prefrontal cortex extending into orbitofrontal cortex.	(92)

*Modified by: Ramdave et al. (81) and Meyer et al. (82)*.

**Table 5B:** Diffusion-tensor images.

**Study objectives**	**Participants**			**Results**	**References**
	**PD ICD/RBDs**	**PD No ICD/RBDs**	**Controls**		
To assess brain white matter tract alterations in PD+ punding vs. controls and PD ICD, and PD non-ICD	PD + Punding	PD Punding –	X	Greater damage of genu of corpus callosum and left pedunculopontine tract in PD-punding vs. PD-without ICD	(93)
To assess brain structural and functional alterations in patients with PD-ICB vs. with controls and PD no-ICB cases.	x	x	x	Cortical thinning in left pre-central and superior frontal cortices, as well as decreased Fractional anisotropy (FA) of the left uncinate fasciculus and parahippocampal tract; increased mean, radial and axial diffusivity of the left parahippocampal tract and right pedunculopontine tract; increased mean and radial diffusivity of the genu of the cingulate cortex and right uncinate fasciculus.	(86)
To determine the changes in DTI associated with medication-related ICD in PD patients undergoing chronic dopamine-replacement therapy.	x	x	x	PD-ICD showed significantly elevated FA in anterior cingulate cortex (ACC), right internal capsule posterior limbs, right posterior cingulum, and right thalamic radiations compared to PD-without ICD	(92)
To identify alterations of white matter tract in drug-naïve PD- ICDs	x	x	x	Decreased connectivity in left and right cortico-thalamic tract, left and right cortico-pontine tract, left and right corticospinal tract, left and right superior cerebellar peduncle and left and right middle cerebellar peduncle between PD-ICD compared to PD-without ICD. Decreased connectivity in left and right inferior longitudinal fasciculus, genu and body of corpus callosum, left and right corticospinal tract, left superior cerebellar peduncle and left and right cingulum in PD-ICD compared to control.	(94)

*Modified by: Ramdave et al. (81) and Meyer et al. (82)*.

**Table 5C:** Resting state and Task-based fMRI.

**Study rationale**	**Participants**			**Ligand**	**Results**	**References**
	**PD ICD/RBDs**	**PD No ICD/RBDs**	**Controls**			
**RESTING-STATE fMRI**						
To identify corticostriatal connectivity (especially between ventral striatum and cortical limbic regions) in PD ICDs	X	X	X	Resting state	Significant functional disconnection between left anterior putamen and both left inferior temporal gyrus and left ACC, in PD-ICD	(95)
To investigate functional alterations in PD ICB+; vs. controls and PD no ICB	X	X	x		Increased functional connectivity of bilateral pre-central and post-central gyrus in PD-without ICDs vs. control and PD-ICD. Increased functional connectivity in left frontoparietal and visual network positively correlated with ICD duration	(86)
To assess whether a functional dysregulation of the habenula and amygdala (modulators of the reward brain circuit), contributes to PD punding	Punding	X	x		Higher functional connectivity of habenula and amygdala with thalamus and striatum bilaterally, and lower connectivity between bilateral habenula and left frontal and pre-central cortices in PD-punding vs. PD-without ICDs and control. Lower functional connectivity between right amygdala and hippocampus in PD-punding vs. PD-without ICD.	(87)
To investigate differences in both affective and sensorimotor striatal circuitries between PD ICD, PD-No ICDS and association with impulsive behavior	X	X	N/A		PD-ICD compared to PD-without ICD: Stronger connectivity between left putamen and central operculum, left caudate and occipital fusiform gyrus and various cerebellar regions, left Globus Pallidus internali and left superior temporal gyrus, left subthalamic nucleus(STN) and left caudate, parietal and temporal areas. Weaker connectivity between left GPe and various frontal cortical areas, left STN and various frontal areas, parietal area and paracingulate, middle frontal gyrus and subcortical areas.	(90)
To investigate brain network connectivity at baseline in a cohort of drug-naive PD patients who successively developed ICDs over a 36-month follow-up period compared with patients who did not.	Drug Naive PD		X		Increased baseline connectivity in subtantia nigra (SN) and decreased baseline connectivity in default mode network and central executive network in PD patients who develop ICD after chronic dopaminergic treatment compared to those who did not	(96)
To investigate intrinsic neural networks connectivity changes in PD with and without ICD.	X	X	x		Increased connectivity in salience network and default mode network and decreased connectivity in central executive network in PD-ICD. Increased connectivity in salience network positively correlated with ICD symptom severity.	(97)
**TASK-BASED fMRI**						
To identify differences in CBF responses to DA in mesocorticolimbic regions in PD patients with and without ICD	X	X	N/A	On/Off state	Increased CBF in bilateral striatum, SN, periaqueductal gray matter, insular cortex, and ventromedial prefrontal cortex in PD-ICD compared to PD-without ICD. Increased CBF in bilateral VS in PD-ICD in ON state vs. OFF state.	(98)
To identify dysfunctional brain reward networks in PD- Dopamine dysregulation síndrome (DDS)	DDS	X	N/A	ON and OFF medication states. Drug-related visual stimuli. Drug Effects Questionnaire	Exposure to drug-cues increase subjective feeling of being “ON” during both “ON” and “OFF” medication scans, which corresponds to significantly increased activation in ventral striatum (VS) in PD-DDS.	(99)
To demonstrate that DA treated PD patients with ICDs have increased functional connectivity between the ventral striatum and components of the limbic striato-pallido-thalamocortical loop and additionally to explore amygdala connectivity with reward network components.	X	X	N/A	Incentive learning task with “gain” and “loss” conditions. ON and OFF medication states	Elevated ventral striatal connectivity to anterior cingulate cortex (ACC), orbitofrontal cortex (OFC), insula, putamen, globus pallidus and thalamus in PD-ICD patients compared to PD-without ICD. No difference in connectivity seen between ON and OFF medication scans. Ventral striatum to subgenual ACC connectivity positively correlated with reward learning performance.	(100)
To demonstrate a link between hypersexuality in PD and increased processing in brain regions linked to sexual motivation and cue reactivity	Hypersexuality (PD-HS)	X	N/A	Visual stimuli presented of sexual, other-reward related and neutral cues. ON and OFF medication states	Increased sexual desire correlated with enhanced activation in VS, cingulate and OFC in PD-HS when ON medication.	(101)
To quantify resting cerebral blood flow (CBF) and blood oxygenation level dependent (BOLD) fMRI to measure neural responses to risk taking during performance on the Balloon Analog Risk Task (BART).	X	X	N/A	Balloon Analog Risk Task	Significantly reduced BOLD activity in right ventral striatum during all risk taking trials and significantly reduced resting CBF in right ventral striatum, in PD-ICD	(102)
To demonstrate that DA would be associated with faster learning from gain outcomes along with greater ventral striatal positive δ activity in PD ICDs vs. PD without ICDs	X	X	X	Probabilistic reward learning task. ON and OFF medication states.	Greater left OFC activity in PD-DD patients compared to PD-without ICD. PD patients in the ON state compared to OFF state learn faster from gain outcomes during the task along with greater ventral striatal activity to unexpected rewards.	(103)
To demonstrate that DA would be associated with greater risk taking and lower ventral striatal activity in PD with ICD vs. PD without ICD	X	X	X	Risk task with “Gain” and “Loss” condition. ON and OFF medication states.	ON state associated with lower bilateral ventral striatal activity compared to the OFF state in patients with ICD with the reverse finding in PD control group. Greater correlation between BOLD activity and risk in PD-ICD compared to PD-without ICD in bilateral ACC and caudate, and left OFC.	(104)

*Modified by: Ramdave et al. (81) and Meyer et al. (82)*.

**Table 5D:** PET and SPECT Studies.

**Study objectives**	**Participants**			**Ligand**	**Results**	**References**
	**PD ICD/RBDs**	**PD No ICD/RBDs**	**Controls**			
**PET**						
To evaluate l-dopa induced dopamine neurotransmission in the striatum of patients with DDS compared with PD control patients.	Dopamine dysregulation syndrome (DDS)	x	N/A	[11C] raclopride (D2/D3-affinity)	Greater reduction in ventral striatal binding potential in DDS (14.4%) vs. control (3.6%). Positive correlation with L-DOPA wanting but not liking	(105)
To investigate the effects of reward-related cues and L-dopa challenge in patients with PD ICD; and PD without ICD on striatal levels of synaptic dopamine	x	x	N/A		Greater reduction in ventral striatal binding potential following task in ICD (16.3%) vs. control (5.8%).	(106)
To compare dopaminergic function during gambling in PD patients, with and without pathological gambling (PG), following dopamine agonists.	PD-PG	x	N/A		Greater reduction in ventral striatal binding potential during task in ICD (13.9%) vs. PD control (8.1%)	(107)
(1) To investigate dopamine neurotransmission in PD patients with multiple ICDs, single ICDs and non-ICD controls in response to reward-related visual cues. (2) To compare clinical features of the above three groups.	Single ICD Multiple ICDs	X	N/A		Greater reduction in ventral striatal binding potential in single (17.19%) and multiple ICD (17.51%) vs. control (6.47%). No significant difference between ICD groups	(108)
To investigate whether ICD in PD are associated with greater D3 dopamine receptor availability	x	x	x	[11C]-(+)-PHNO (D3-affinity	Greater reduction (20%) in ventral striatal binding potential in ICD vs. non-ICD.	(109)
To investigate the role of extrastriatal dopaminergic abnormalities in PD patients with PG	Gambling (PD-PG)	X	N/A	[11C] FLB-457 (Extra- striatal D2/D3 affinity)	Greater reduction in midbrain binding potential in PG vs. control during gambling. Increase in binding potential in ACC in PG vs. control in control task	(110)
To investigate the possible involvement of the mesostriatal and mesolimbic monoaminergic function in ICDs associated with PD	x	X	N/A	[18F] F-Dopa	Increased binding potential (35%) in medial orbitofrontal cortex in ICD vs. control PD without ICD.	(111)
To investigate DA-induced changes in brain activity that may differentiate patients with PD with DA-induced PG) from PD without PG	PD-Gambling (PG)	X		H2(15)O [Regional cerebral blood flow (rCBF)]	Significant reduction in rCBF in left lateral orbitofrontal cortex, right rostral cingulate zone, right amygdala, left ventral anterior external pallidum in PG, while controls showed increased rCBF in these areas for ON vs. OFF phase scans.	(112)
To investigate the extrastriatal dopaminergic neural changes in relation to the medication-related ICDs in PD.	X	x	x	[18F]FP-CIT (DAT density/PET)	Increased binding potential in right ventromedial prefrontal cortex, left insular and right posterior cingulate cortex and reduced binding potential at left nucleus accumbens, ventral striatum and ventral pallidum, in ICD vs. non-ICD.	(113)
To describe the metabolic PET substrate and related connectivity changes in PD ICDs.	X	X	N/A	[18F] FDG	Increased glucose metabolism in right middle and inferior temporal regions in PD-ICD compared with PD-CNTR. Higher metabolism in these areas in patients with multiple ICDs vs. single ICD	(114)
**SPECT**						
To investigate resting state brain perfusion in PD patients with active PG compared with PD controls and healthy controls.	PG X	X	X	[123I]FP-CIT (DAT density/SPECT	Reduced DAT binding in right ventral striatum (nucleus accumbens) of PD-PG compared to PD-CNTR	(115)
To assess presynaptic dopaminergic function	X	X	x		Reduced tracer binding in the ventral striatum of PD patients with PG compared to PD controls	(116)
To assess striatal dopamine transporter (DAT) density in PD ICD	X	X	N/A		Lower DAT binding in right striatum with trend in ICD.	(117)
To follow-up data from medication-naïve PD patients who underwent dopamine transporter SPECT imaging at baseline and were subsequently treated with DA replacement therapy.	PD-Drug Naïve and subsequently treated with dopaminergic therapy		N/A	[123I]FP-CIT (DAT density/SPECT	11 patients developed ICD symptoms after DRT. PD-ICD patients had lower DAT availability in right ventral striatum, anterior-dorsal striatum and posterior putamen compared to control	(118)
To assess cortico-striatal connectivity in PD ICDs	X	X	N/A		Significant reduction in tracer uptake in left putaminal and left inferior frontal gyrus in PD-ICD vs. PD without ICDl.	(119)
To investigate resting state brain perfusion in PD PG compared with matched PD controls and healthy controls.	X	X	X	99mTc-ECD (rCBF/SPECT)	PD-PG showed a disconnection between the ACC and the striatum, which was not observed in PD patients without PG and HC groups.	(115)

*Modified by: Ramdave et al. (81) and Meyer et al. (82)*.

## Structural and Functional Magnetic Resonance Imaging

1. Structural MRI changes have been reported in PD patients with ICDs with a selective atrophy in the orbitofrontal and anterior cingulate cortices (areas involved in behavioral modulation). Atrophy in the orbitofrontal cortex has been reported in PD patients with ICDs (85, 91).
2. Functional brain resonance (fMRI) studies have reported an abnormal metabolism on the frontostriatal and cingulate cortices, the nucleus accumbens and the amygdala (2, 120).
3. A connectivity dysfunction between the striatal and limbic areas has been proposed. Brain connectivity was impaired in PD patients with ICDs with respect to the PD individuals without ICDs involving the neurocognitive network. A decreased connectivity has been identified in the central executive networks (mediofrontal areas, anterior cingulate and para-cingulate cortices), while an increased connectivity has been identified in the salience network (limbic-paralimbic network) and in the default mode network (pre-cuneus and posterior cingulate, bilateral inferior-lateral-parietal and ventromedial frontal cortices) (95, 97).

Single photon emission computed tomography (SPECT) of the dopamine transporter (DAT).

DAT regulates dopamine turnover. A reduced DAT binding in PD patients with PG and ICDs has been identified in PD patients with ICD compared to PD patients without ICD or healthy controls. This reduced binding of DAT has been suggested as a potential biomarker for risk of developing ICD symptoms (2, 36, 60). The binding reduction was not uniformly reproduced in different studies: some reported a reduction in right ventral striatum (2, 102), while others in the left putamen and left inferior frontal gyrus. These data could reflect a mesolimbic projection and frontostriatal disconnection, suggesting a vulnerability or maladaptive synaptic plasticity under non-physiological DA stimulation (2).

## Positron Emission Tomography (PET) With 11C-raclopride

Positron emission tomography (PET) neuroimaging with 11C-raclopride explores the DA fluxes within the basal ganglia. The 11C-raclopride is a reversible binding to the post-synaptic D2/3 receptor that competes with endogenous DA (2, 8, 22, 106, 107). Decreased 11C-raclopride binding is an indirect measure of increased endogenous dopamine release or “hyperdopaminergic state.”

A significant reduction of 11C-raclopride binding has been reported in ventral striatum, but not in dorsal striatum, in PD with ICDs (single or multiple) as compared to PD individuals without ICDs, following generic reward-related vs. neutral visual stimuli.

A more selective radioligand [18F]fallypride, with high affinity D2-like receptors (D2/D3 receptors) confirmed a reduced binding within the VS and putamen (121).

All of these findings contribute to support a mesocorticolimbic imbalance in PD with ICDs (108).

## PD- ICDs Treatment

The first approach for ICD is prevention, and a key element is patient and family education concerning potential risks of different dopaminergic therapies. Physicians should be aware of predisposing risk factors and balance cost/benefit before DA prescriptions, excluding genetic factors and taking into consideration clinical findings, such as young age, early PD onset, lengthy disease duration, personal history of addictive behaviors, male gender, short-acting DA drugs, behavior and mood disorders (apathy, depression), DBS and certain cultural factors that require attention before prescription.

When ICDs appear, treatment continues to be a challenge. Individualized treatment must be conducted, identifying potential variables, such as motor status, comorbidities, other non-motor symptoms and quality of life (27, 122, 123).

The relevance of prevention is supported by NICE guidance that includes written information, or verbal information recorded in writing, at DA initiation of treatment. The authors emphasize the relevance of communicating to patients, relatives and carers the risk of ICDs due to the potential impact on their lives and for early detection (124).

The first approach for the treatment of ICD symptoms is the reduction or discontinuation of DAs. However, it should be considered that neuropsychiatric traits may persist for at least 12 weeks after drug withdrawal (60, 61, 123).

Nonetheless, in certain cases this strategy is not feasible, and some patients are at risk of developing DA withdrawal syndrome and worsening motor symptoms (21, 61, 123).

Although animal PD models have identified serotonin (5HT) depletion as a higher risk for impulsivity and risk behaviors, the serotonin reuptake inhibitors (SSRIs) used to treat ICDs had controversial results (22, 123).

Atypical antipsychotics, such as clozapine and quetiapine have been used to treat ICDs in PD, but no randomized trials have been conducted and evidence is limited (2, 7).

Taking into consideration that specific SNP opioid receptors have been identified as stronger risk factors for ICDs, opioid antagonists employed in the treatment of PG have produced controversial results (naltrexone, nalmefene) (2, 7, 16, 22, 60, 123).

A number of drugs administered to increase Gabaergic inhibition (valproate, topiramate), as well as new drugs to preserve ventral striatal DA system (zonisamide, donepezil, noradrenaline reuptake inhibitor) have been essayed (2).

As previously mentioned, controversial data are available concerning DBS and ICD treatment. A favorable response through reduction in dopaminergic requirements has been noted. It has been suggested that STN stimulation could reduce the risk for ICDs by increased reward-driven behaviors by inhibitor effect in the indirect dopaminergic pathway. However, some patients may develop transient de novo ICDs after STN DBS, and selective patients may develop ICDs a long time after DBS (123, 125).

A non-pharmacologic approach includes cognitive behavioral therapy and patient and caregiver education (7, 60).

## Conclusions

The treatment used for PD, particularly DA, is associated with the development of ICDs and related behaviors. Susceptibility to these disorders depends on the associated risk factors.

ICDs can have serious personal, family, psychosocial, financial, and medical consequences. However, in contrast, artistic activities have been described in patients with PD while undergoing treatment with DA. These patients are compulsive but report a positive influence on quality of life.

These findings highlight the need for a very critical approach at the moment of Dopaminergic Replacement therapy choice.

## Author Contributions

EG: study concept, design, and editing. VA: study concept and editing of manuscript.

### Conflict of Interest Statement

The authors declare that the research was conducted in the absence of any commercial or financial relationships that could be construed as a potential conflict of interest.
